# Metal Duo Damages Lungs: Lead and Manganese in Fine Particulates

**Published:** 2007-03

**Authors:** Bob Weinhold

Extensive evidence indicates that fine particulates can damage human lungs. But much remains unknown about exactly which components of these particulates are to blame. In a small study of Korean children, researchers have found that two metals, lead and manganese, are among the substances likely at fault **[*EHP* 115:430–434; Hong et al.]**.

To pin down the particulate culprits, the researchers evaluated 43 children who attended school on an island near Incheon City. The island has low traffic density and industrial emissions, but concentrations of fine particulates 2.5 μm in diameter or smaller were relatively high by U.S. standards, perhaps owing to natural sources or dust from China or Mongolia. The mean of 20.27 μg/m^3^ measured during the six-week study period was about one-third higher than the U.S. annual standard.

After an introductory period during which the children (median age 10) learned how to use a peak expiratory flow meter to measure their lung function, each child used the device at three fixed times every day. Meanwhile, the researchers sampled fine particulates every day on the roof of a building 2 km from the school and analyzed the concentration of five metals: aluminum, iron, lead, manganese, and zinc. Previous studies have shown these metals might play either beneficial or harmful roles when present in particulates.

The researchers also tested the children for polymorphisms of *GSTM1* and *GSTT1.* These two genes play a role in the function of the enzyme glutathione *S*-transferase, which scavenges the damaging reactive oxygen species created by some metals. In addition, they took into account many other factors, including weather, day of the week, sex, age, height, weight, asthma history, passive smoking exposure at home, and socioeconomic status. They did not test for other metals, acquire data on other lung-damaging pollutants (such as nitrogen dioxide or ozone), or measure personal fine particulate exposures.

Typical of Asian populations, roughly half the children did not have one gene or the other due to deletion. The team found that lead and manganese were linked with significant reductions in peak expiratory flow rate, regardless of whether a child had either of the tested gene polymorphisms. The three other metals had no significant effects, even though they sometimes were present at much higher concentrations than lead and manganese. The team acknowledges that additional studies are needed to comprehensively determine the impact of metals on the respiratory system.

## Figures and Tables

**Figure:**
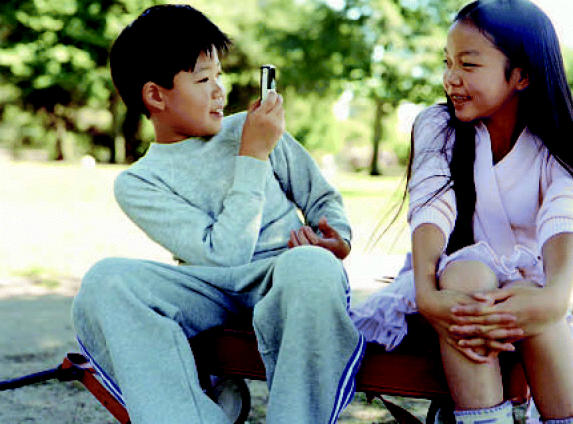
Gotcha! In a study of Korean children scientists identified some of the health-damaging components of fine particulates.

